# Clinical characteristics and prognosis of exchange transfusion in infants with severe pertussis

**DOI:** 10.1186/s12887-025-06132-3

**Published:** 2025-10-03

**Authors:** Pingping Liu, Zhenghui Xiao, Jiaotian Huang, Yanying Chen, Juan Liu

**Affiliations:** Department of Emergency & Key Laboratory of Pediatric Emergency Medicine of Hunan Province, The Affiliated Children’s Hospital of Xiangya School of Medicine, Central South University (Hunan Children’s Hospital), Changsha, Hunan China

**Keywords:** Severe pertussis, Infants, Exchange transfusion, Prognosis

## Abstract

**Background:**

To explore the efficacy and clinical significance of exchange transfusion (ET) therapy in infants with severe pertussis, and provide a basis for the diagnosis and treatment of severe pertussis.

**Methods:**

45 infants diagnosed with severe pertussis and receiving ET treatment in the intensive care unit of Hunan Children’s Hospital from January 1, 2019 to June 30, 2024 were selected as the study subjects. According to the prognosis, they were divided into surviving group (*n* = 35) and mortality group (*n* = 10), and the clinical manifestations and biochemical indicators of the two groups were compared. The comparison between groups of count data was conducted using the chi square test. The *t*-test is used to compare between groups whose measurement data conforms to a normal distribution; The Mann Whitney *U* test is used to compare between groups that do not follow a normal distribution.

**Results:**

The mortality of 45 infants with severe pertussis who received ET treatment was 22.2% (10/45). The incidences of oliguria, bradycardia, and pleural effusion in the mortality group were higher than those in the surviving group, and the differences were statistically significant (*P* < 0.05). The white blood cells (WBC) and neutrophils (NE) in mortality group were significantly higher than those in surviving group before ET treatment. After 24 h and 48 h of ET treatment, the WBC and NE in mortality group were still higher than those in surviving group (*P* < 0.05). There were no statistically significant differences in WBC and NE between the two groups 72 h after ET treatment (*P* > 0.05). The levels of C-reactive protein (CRP), procalcitonin (PCT), N-terminal B-type natriuretic peptide precursor (NT-BNP), creatinine (Cr), lactate dehydrogenase (LDH), creatine kinase (CK), and myoglobin (MYO) in mortality group were all higher than those in surviving group before ET therapy (*P* < 0.05). The PH and PO_2_ levels in the blood gas analysis of mortality group were lower than those of surviving group before ET treatment, while PCO_2_ and lactate (Lac) levels were higher than those of mortality group (*P* < 0.05). The incidences of pulmonary hypertension, heart failure, cardiogenic shock, acute kidney injury, and pertussis encephalopathy in mortality group were higher than those in surviving group, and the differences were statistically significant (*P* < 0.05). The usage rates of hormones and vasoactive drugs in mortality group were higher than those in surviving group (*P* < 0.05).

**Conclusions:**

ET is an important treatment for improving the prognosis of infants with severe pertussis. Infants with severe pertussis who undergo ET have a higher peak WBC count, and those with concomitant pulmonary arterial hypertension, cardiac, renal, neurological dysfunction have a poorer prognosis. Early intervention and active treatment are necessary.

## Background

Pertussis is an acute and highly contagious respiratory disease caused by bordetella pertussis. Pertussis remains one of the leading causes of death from infectious diseases in infants, especially in infants who have not yet started vaccination [1]. Previous clinical data reported that the incidence of pertussis admitted to the intensive care unit (ICU) is approximately 2.1–18.6 per 100,000 infants, and the mortality of pertussis after ICU treatment is about 4.8% [2]. According to our domestic hospital reports, there is no exact incidence of severe pertussis in China, but almost all cases are infants [3]. The treatment of severe pertussis mainly involves supportive therapy, including mechanical ventilation (conventional or high-frequency), inhalation of nitric oxide (NO), exchange transfusion (ET), and extracorporeal membrane oxygenation (ECMO). Among them, ET has been reported as a method to reduce the white blood cell (WBC) load and improve the prognosis of infants [4]. Recently, Wu et al. revealed ET could effectively reduce peripheral blood leukocytes in infant pertussis with extreme leukocytosis [5]. Currently, there are relatively few reports on ET for severe pertussis both domestically and internationally [6]. This study takes 45 infants diagnosed with severe pertussis and treated with ET in Hunan Children’s Hospital from January 1, 2019 to June 30, 2024 as the research subjects. Statistical methods such as chi-square test and *t/U* test were used to analyze their clinical characteristics and prognostic factors to provide a basis for the treatment of severe pertussis.

## Methods

### Study subjects

We employed a retrospective study design to analyze a total of 45 infants diagnosed with severe pertussis and treated with ET in the ICU of Hunan Children’s Hospital from January 1, 2019 to June 30, 2024. They were divided into the surviving group (*n* = 35) and the mortality group (*n* = 10) based on their prognosis. The diagnosis of pertussis was confirmed by positive pertussis nucleic acid test, with specimens collected from endotracheal aspirates during invasive ventilation or bronchoalveolar lavage fluid obtained through bronchoscopy. Severe pertussis was defined as the presence of one or more of the following: recurrent apnea, hypoxemia, pertussis encephalopathy, or cardiovascular dysfunction. Inclusion criteria: Patients who met the diagnostic criteria for severe pertussis and received ET treatment. Exclusion criteria: Patients who do not meet the diagnostic criteria for severe pertussis and who have not received ET treatment. Indications for ET included a total WBC count ≥ 48 × 10^9^/L and a lymphocyte count ≥ 15 × 10^9^/L; or a total WBC count ≥ 25 × 10^9^/L and a lymphocyte count ≥ 15 × 10^9^/L, with an increase of ≥ 50% within 24 h [4].

### Treatment methods

General information such as gender, age in months, year of onset, disease course, and vaccination status of the infants was collected. Clinical symptoms and signs were recorded, including respiratory symptoms (paroxysmal cough, posttussive vomiting, apnea, wheezing, and shortness of breath), cardiac symptoms (tachycardia and bradycardia), renal symptoms (oliguria and edema), neurological symptom (convulsion), and other symptoms (cyanosis and fever). Laboratory indicators such as routine complete blood count, liver and kidney function, myocardial enzymes, arterial blood gas, and lactate were collected during hospitalization. Chest imaging features were recorded, and pulmonary hypertension (PH) was evaluated by transthoracic echocardiography, with estimation based on the tricuspid regurgitation velocity. Transthoracic echocardiography exams were conducted by a team of two sonographers trained in clinical cardiology and echocardiography, utilizing a standardized imaging technique. Each echocardiogram was subsequently analyzed by a group of three experienced cardiologists possessing expertise in echocardiography. ET method: Before the procedure, tests such as blood transfusion four items, hepatitis panel, and hepatitis B panel were completed. ET was performed after obtaining informed consent from the parents. The total volume of blood exchange was 150–180 mL/(kg·session), with the replacement blood being the same type of red blood cells and plasma, with a ratio of 2:1. The input and output blood speeds were synchronized, and the double-channel blood exchange was carried out at a uniform speed. During the procedure, heart rate, respiratory rate, blood pressure, and transcutaneous oxygen saturation were monitored. After the procedure, a slow intravenous push of calcium gluconate injection was given. Glucocorticoid therapy method: prednisone, 1 mg per kilogram of body weight, administered in divided doses every 12 h, for a treatment course of 7 to 10 days. This study was approved by the Medical Ethics Committee of Hunan Children’s Hospital (Approval No.: HCHLL-2024-322).

### Statistical analysis

Data were processed using SPSS 26.0 statistical software. Categorical variables were described as n (%), and the χ2 test was used for comparison between groups. Continuous variables that followed a normal distribution were expressed as mean ± standard deviation, and the t-test was used for comparison between groups; those that did not follow a normal distribution were expressed as median and interquartile range (IQR), and the Mann-Whitney *U* test was used for comparison between groups. A *P* value < 0.05 was considered statistically significant.

## Results

### Comparison of general data

**Fig. 1:**
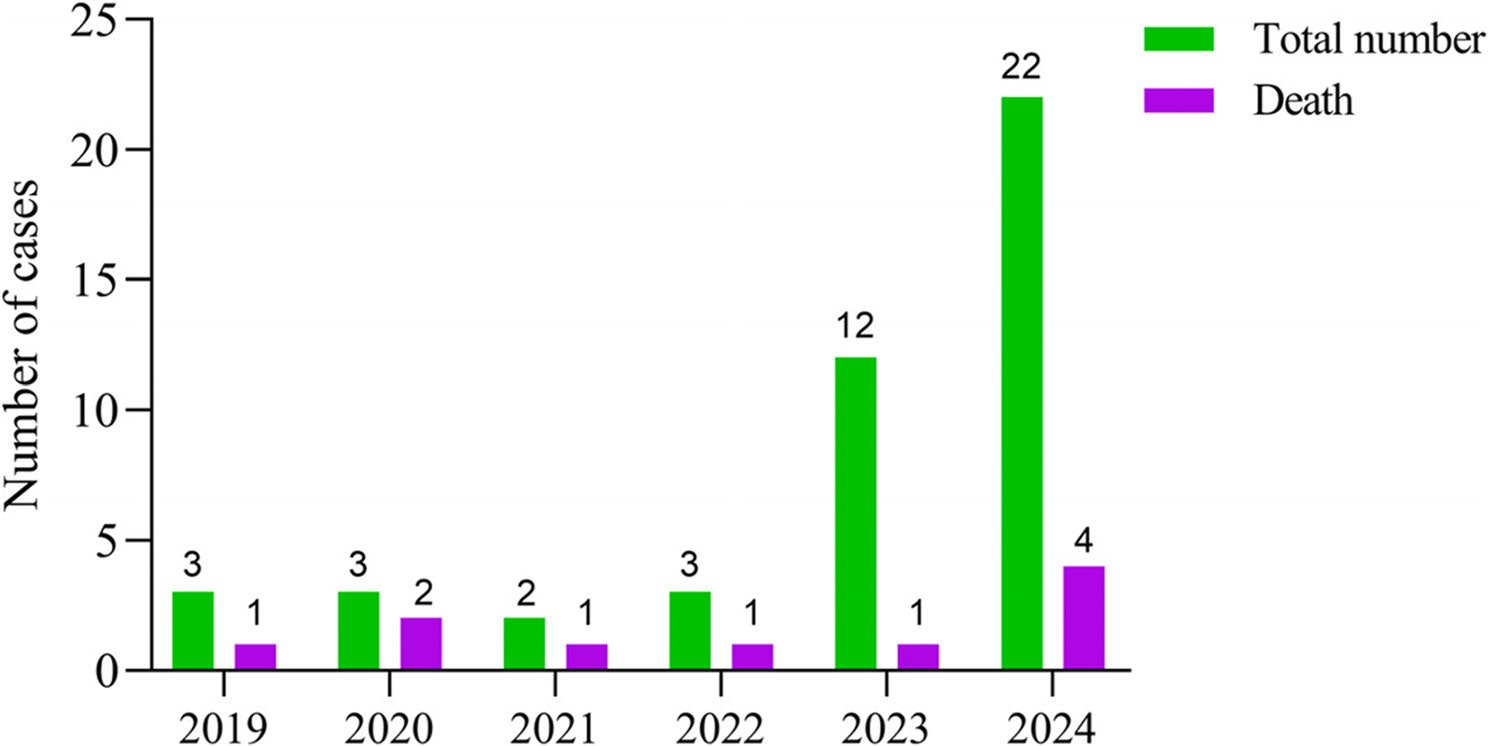
Total number of severe pertussis cases and deaths treated with ET from 2019 to 2024

The mortality of 45 infants with severe pertussis who received ET was 22.2% (10/45). The number of pertussis cases treated with ET in our center in 2023 and 2024 increased significantly compared to previous years, while the mortality decreased significantly compared to the period from 2019 to 2022 (Fig. 1). The median duration of illness for all infants with pertussis who developed severe cases was 10 days, and the average hospitalization cost was 54,353 (30,088 − 79,598). There were no statistically significant differences in gender, age, weight, and duration of illness between the surviving group and the mortality group (P > 0.05). The hospitalization cost and length of stay in the surviving group were higher than those in the mortality group (P < 0.05) (Table 1).

**Table 1:** Analysis of clinical characteristics of infants in different groups

Indicators	Surviving group (*n* = 35)	Mortality group (*n* = 10)	U/χ^2^	*P*
Gender/Male	19 (54.3)	5 (50)	0.057	1
Age/Months	2.0 (1.0, 5.25)	3.0 (1.0, 5.0)	170.00	0.890
Weight/kg	6.0 (4.3, 7.5)	4.9 (4.5, 6.4)	152.50	0.539
Course of illness/days	10.0 (9, 15.0)	10.0 (6.0, 20.0)	149.90	0.479
Length of hospital stay	25 (18, 31)	4 (2, 8)	4.00	0.000
Hospitalization expenses	62,172 (44573, 80643)	26,863 (20828, 69626)	92.00	0.023
Spasmodic cough	34 (97.1)	10 (100)	0.292	1
Posttussive vomiting	20 (57.1)	3 (30)	2.293	0.165
Apnea	5 (14.3)	2(20)	0.193	1
Cyanosis	25 (71.4)	9 (90)	1.452	0.409
Fever	24 (68.6)	10 (100)	4.160	0.089
Convulsion	5 (14.3)	2 (20)	0.193	1
Shortness of breath	33 (94.3)	10 (100)	0.598	1
Gasping for breath	20 (57.1)	6 (60)	0.026	1
Edema	15 (42.9)	8 (80)	4.294	0.071
Oliguria	8 (22.9)	79 (70)	7.779	0.009
Conjunctival hemorrhage	4 (11.4)	2 (20)	0.495	0.601
Bradycardia	5 (14.3)	5 (50)	5.740	0.029
Tachycardia	34 (97.1)	10 (100)	0.292	1
Co-infection	31 (88.6)	9 (90)	0.016	0.899
Pneumothorax	0 (0)	1 (10)	3.580	0.222
Pulmonary consolidation	23 (65.7)	7 (70)	0.064	1
Pleural effusion	1 (2.9)	3 (30)	7.075	0.030

### Comparison of clinical features

Two cases in the surviving group and one case in the mortality group were vaccinated against pertussis, and the difference was not statistically significant (χ2 = 0.305, *P* > 0.05). The incidences of oliguria, bradycardia, and pleural effusion were higher in mortality group than those in surviving group, and the differences were statistically significant (*P* < 0.05). There were no statistically significant differences in other symptoms, signs, and clinical features between surviving group and mortality group (Table 1).

### Comparison of blood routine, biochemical indicators and complications

Before ET, the WBC and neutrophil (NE) counts in the mortality group were significantly higher than those in the surviving group. At 24 and 48 h after ET, the WBC and NE counts in mortality group were still higher than those in surviving group (*P* < 0.05). At 72 h after ET, there was no significant difference in WBC and NE counts between the two groups (*P* > 0.05). There was no significant difference in lymphocyte (LY), hemoglobin (HB) before and after ET between surviving group and mortality group. There was no significant difference in platelet (PLT) before and 24 h after ET in mortality group (*P* > 0.05), but at 48 and 72 h after ET, the PLT in mortality group was significantly lower than that in surviving group (*P* < 0.05) (Table 2). Before ET, the C-reactive protein (CRP), procalcitonin (PCT), N-terminal pro-B-type natriuretic peptide (NT-BNP), creatinine (Cr), lactate dehydrogenase (LDH), creatine kinase (CK), and myoglobin (MYO) in mortality group were all higher than those in surviving group (*P* < 0.05). There were no significant differences in blood urea nitrogen (BUN), albumin (ALB), and creatine kinase isoenzyme (CKMB) between two groups. Before ET, the pH and PO_2_ in mortality group were lower than those in surviving group, while PCO_2_ and lactate (Lac) were higher than those in surviving group (*P* < 0.05) (Table 3). The incidences of pulmonary hypertension, heart failure, cardiogenic shock, acute kidney injury and pertussis encephalopathy in mortality group were higher than those in surviving group, and the differences were statistically significant (*P* < 0.05) (Table 4).

**Table 2:** Analysis of blood routine-related indicators before and after ET in infants of different groups

Complete blood count	Surviving group (*n* = 35)	Mortality group (*n* = 10)	U/χ^2^	*P*
Before ET WBC/×10^9^/L ((4.3–14.2)×10^9^/L)	54.09(49.34, 68.33)	88.91(49.7, 92.22)	101.00	0.043
After ET 24 h WBC/×10^9^/L	30.41(23.96, 35.29)	42.29(34.29, 66.14)	48.00	0.004
After ET 48 h WBC/×10^9^/L	31.15(24.98, 38.00)	48.42(33.79, 53.11)	31.00	0.021
After ET 72 h WBC/×10^9^/L	31.19(24.64, 33.96)	34.18(24.0, 35.06)	21.00	0.782
Before ET NE/×10^9^/L ((0.6–7.5)×10^9^/L)	21.36(13.09, 25.56)	33.79(25.86, 55.98)	58.00	0.001
After ET 24 h NE/×10^9^/L	14.50(9.18, 21.50)	26.06(22.17, 45.39)	44.00	0.003
After ET 48 h NE/×10^9^/L	19.35(12.85, 23.25)	25.86(24.06, 33.58)	30.00	0.019
After ET 72 h NE/×10^9^/L	16.93(11.47, 21.94)	21.09(16.11, 22.91)	25.00	0.502
Before ET LY/×10^9^/L ((2.4–9.5)×10^9^/L)	26.25(19.90, 36.32)	28.40(23.65, 41.30)	146.00	0.429
After ET 24 h LY/×10^9^/L	9.92(7.95, 12.48)	10.90(8.58, 15.70)	116.50	0.463
After ET 48 h LY/×10^9^/L	9.72(8.27, 12.00)	9.04(6.14, 14.21)	69.00	0.449
After ET 72 h LY/×10^9^/L	9.05(7.01, 10.52)	6.07(3.81, 6.89)	13.00	0.140
Before ET HB/g/L (97–183 g/L)	100(90, 113)	93(89, 114)	154.50	0.575
After ET 24 h HB/g/L	109(96, 126)	111(79, 161)	133.50	0.839
After ET 48 h HB/g/L	103(91, 122)	118(110, 149)	36.70	0.051
After ET 72 h HB/g/L	100(90, 122)	113(99, 117)	25.50	0.523
Before ET PLT/×10^9^/L ((183–614)×10^9^/L)	663(611, 744)	615(533, 788)	118.00	0.120
After ET 24 h PLT/×10^9^/L	117(106, 145)	81(65, 181)	96.00	0.371
After ET 48 h PLT/×10^9^/L	180(133, 220)	75(53, 152)	28.50	0.016
After ET 72 h PLT/×10^9^/L	230(170, 291)	50(32, 56)	25.60	0.022

**Table 3:** Analysis of biochemical indicators of infants in different groups before ET

Organ function indicators	Surviving group (*n* = 35)	Mortality group (*n* = 10)	U/χ^2^	*P*
CRP/mg·L^−1^ (0–5)	28.44 (8.48, 50.94)	100.12 (50.94, 138.55)	73.00	0.007
PCT/ng·mL^−1^ (0-0.05)	0.60 (0.13, 2.38)	5.08 (0.3, 22.99)	73.50	0.038
NT-BNP/pg·mL^−1^ (0-236)	1127.5 (357.6, 3891.2)	56,732 (36578, 71722)	21.00	0.000
BUN/mmol·L^−1^ (2.5–6.5)	3.49 (2.17, 4.61)	3.04 (2.44, 3.62)	152.00	0.530
Cr/µmol·L^−1^ (19–44)	31.50 (27.52, 35.45)	45.00 (25.00, 76.00)	85.50	0.036
LDH/IU·L^−1^ (180–430)	504.5 (385, 658)	1842 (547, 3850)	68.00	0.012
ALB/g·L^−1^ (39–54)	40.45 (35.67, 43.22)	39.1 (37.5, 39.8)	130.00	0.219
CK/U·L^−1^ (0-225)	160.45 (126, 321)	434 (281, 689)	87.00	0.049
CKMB/U·L^−1^ (0–50)	31.16 (24.1, 45.12)	38.1 (26.4, 54.03)	142.00	0.368
MYO/ng·mL^−1^ (0–70)	64 (42.2, 148.9)	269.1 (108.4, 611.9)	56.00	0.003
PH (7.35–7.45)	7.35 (7.29, 7.39)	6.95 (6.84, 7.15)	14.00	0.000
PO_2_/mmHg (80–100)	64 (60, 76)	51 (35, 72)	66.50	0.003
PCO_2_/mmHg (35–45)	63 (52, 81)	86 (76, 100)	41.50	0.000
Lac/mmol·L^−1^ (0.44–1.78)	2.0 (1.3, 2.7)	2.8 (2.2, 12.1)	39.00	0.000

**Table 4:** Analysis of complications in infants of different groups

Indicators	All patients (*n* = 45)	Surviving group (*n* = 35)	Mortality group (*n* = 10)	χ^2^	*P*
Respiratory failure	32 (71.1)	22 (62.9)	10 (100)	5.223	0.022
Pulmonary hypertension	14 (31.1)	6 (17.1)	7 (60)	10.578	0.003
Coagulation dysfunction	25 (55.6)	19 (54.3)	6 (60)	0.918	0.338
Heart failure	16 (35.5)	7 (20)	9 (90)	16.633	0.000
Cardiogenic shock	12 (26.6)	4 (11.4)	8 (80)	18.701	0.000
Acute kidney injury	8 (17.8)	3 (8.6)	6 (60)	12.857	0.000
Pertussis encephalopathy	7 (15.6)	3 (8.6)	4 (40)	5.849	0.034

### Comparison of treatment and prognosis between the two groups

There were no differences between the two groups of infants in terms of the initiation of pertussis treatment and the choice of antibiotics. The usage rates of hormones and vasoactive drugs in the mortality group were higher than those in the surviving group (*P* < 0.05). Due to the critical condition upon admission, 4 infants in mortality group did not receive treatment to lower pulmonary artery pressure (*P* < 0.05). A total of 3 infants received ECMO treatment. One child was successfully treated with ECMO, one child died after treatment was abandoned due to severe ARDS and prolonged lung recovery time complicated by adenovirus infection, and one child died due to ECMO complications (cerebral hemorrhage) (Table 5).

**Table 5:** Analysis of treatment and prognosis of infants in different groups

Indicators	Surviving group (*n* = 35)	Mortality group (*n* = 10)	U/χ^2^	*P*
Azithromycin	30 (85.7)	6 (60)	3.214	0.073
Erythromycin	12 (35.3)	5 (50)	0.705	0.401
Sulfonamide	18 (51.4)	4 (40)	0.407	0.524
Gamma globulin	7 (20)	5 (50)	3.580	0.101
Glucocorticoid	5 (14.3)	8 (80)	16.350	0.000
Drugs for reducing pulmonary artery pressure	2 (5.7)	6 (60)	17.127	0.001
Vasoactive drugs	5 (14.3)	9 (90)	23.008	0.000
Invasive respiratory support	23 (65.7)	10 (100)	4.675	0.101
Blood purification	7 (20)	5 (50)	3.580	0.058
ECMO	1 (2.9)	2 (20)	3.673	0.119

## Discussion

In recent years, the incidence of pertussis has significantly increased in some countries and regions, including China, and severe pertussis has become one of the main causes of death related to bacterial infections in infants in pediatric ICU [7, 8]. Studies have shown that the younger the child, the more likely they are to progress to severe pertussis, especially infants under 3 months of age, who may require closer monitoring in the ICU [9, 10]. In this study, the median age of surviving group was 2 months, and that of the mortality group was 3 months, with no statistically significant difference. This is consistent with the reported age of pertussis onset, especially severe pertussis, both domestically and internationally, which may be related to the fact that infants under 3 months of age have not yet been vaccinated and have low immunity. In China, infants need to receive one dose of pertussis vaccine at 3, 4 and 5 months of age respectively. Additionally, the symptoms of small infants after infection are atypical, making it easy to misdiagnose or miss the diagnosis, which may eventually lead to severe pertussis or even death. Moreover, there were no differences between survival and mortality group in terms of gender, weight, and the number of days it took to develop severe illness. However, the overall hospitalization costs and duration of the surviving group were higher than those of the mortality group, possibly because infants in the mortality group often died during the acute phase of the disease, while those in the surviving group required more time for treatment after being transferred out of the ICU. To date, there are no unified guidelines or standards for the diagnosis and treatment of severe pertussis. In China, the definition of severe pertussis is often based on different organ dysfunctions, such as recurrent apnea, hypoxemia, pertussis encephalopathy, and cardiovascular dysfunction. A recent multicenter study from Turkey showed the the mortality rate for severe pertussis was 8% [11]. Another domestic study indicated that the mortality rate for children with severe pertussis is 22.35% [12]. In this study, the mortality of 45 infants with severe pertussis who received ET treatment was 22.2%. The mortality is similar to that of this domestic study. The number of cases of pertussis treated with exchange transfusion in our center in 2023 and 2024 has significantly increased compared to previous years, while the mortality decreased significantly compared to the period from 2019 to 2022.

Studies have shown that cyanosis, fever, dyspnea, pulmonary rales, wheezing, respiratory rate > 21 breaths/min, heart rate > 180 beats/min, concurrent infection, pneumonia, etc. are prone to progress to severe pertussis [13–15]. During hospitalization, an average heart rate ≥ 115 beats/min and apnea are risk factors for pertussis death [16, 17]. In this study, all infants presented with a certain proportion of paroxysmal cough, crowing-like ending, posttussive vomiting, apnea, cyanosis, fever, and convulsions, with no statistically significant differences. However, the proportion of oliguria, bradycardia, and pleural effusion in mortality group were higher than those in surviving group. Decreased heart rate and oliguria indicate high-risk signals such as respiratory, cardiac, and renal insufficiency [18]. Research has found that leukocytosis is one of the risk factors for death in severe pertussis. ET therapy is considered the main method to reduce the WBC load in pertussis and can improve prognosis [19, 20]. However, despite comprehensive intensive care measures, the mortality of severe pertussis remains as high as 40–75% [21, 22]. ET is a safe and mature treatment method routinely performed in the ICU, which can not only remove the aggregated WBC in the blood vessels but also replace the pertussis toxin in the circulation. Some studies suggested that ET should be performed before organ failure or hypotensive shock to demonstrate its therapeutic value [6]. Recent studies have indicated that when infants with severe pertussis have a body temperature ≥ 38.5 °C, CRP > 30 mg/L, and WBC > 40.0 × 10^9^/L, ET should be considered. Relevant reports have found that the overall survival rate of pertussis patients treated with ET is 67.9% [23]. In this study, the indications for ET were as described in the methodology section, mainly based on WBC level, disease progression speed, and organ function. Before ET, the WBC and NE in mortality group were significantly higher than those in surviving group. After 24 h and 48 h of ET, the WBC and neutrophils in mortality group were still higher than those in surviving group. At 72 h, there were no statistically significant differences in WBC and neutrophils between two groups. Before and after ET, there were no statistically significant differences in lymphocytes and hemoglobin between surviving group and mortality group. However, the platelets in the mortality group were significantly lower than those in the surviving group at 48 h and 72 h after ET. ET therapy could cause a certain degree of platelet decline in the short term [24]. The decreased platelet count was closely related to the severity of pertussis [25]. Therefore, we speculated that the lower the platelet count after ET therapy, the poorer the prognosis may be. The overall survival rate of the 45 infants with pertussis treated with ET in this study was 77.8%, which was higher than the current relevant reports, suggesting that ET plays an important role in improving the prognosis of infants with severe pertussis.

Before ET, CRP and PCT levels in the mortality group were higher than those in the surviving group, suggesting that the immunity of pertussis patients was weakened and their susceptibility to pathogens increased. When combined with other bacterial or viral infections, pertussis becomes more severe [26]. Pulmonary hypertension is a risk factor for death in severe pertussis, with up to 65% of pertussis deaths being associated with pulmonary hypertension [27]. Pertussis-induced pulmonary hypertension may be related to high WBC level, high reactivity of pulmonary vessels to hypoxia, and the effect of pertussis toxin on vascular smooth muscle [28, 29]. Although the incidence of pertussis encephalopathy is relatively low, when clinical manifestations such as consciousness disorders or limb rigidity and spasms occur, pertussis encephalopathy should be suspected. Its pathogenesis is considered to be related to the destruction of the integrity of the blood-brain barrier by pertussis toxin [30]. Therefore, early identification and intervention of pertussis encephalopathy are crucial for improving the prognosis of infants. In this study, the incidences of pulmonary hypertension and pertussis encephalopathy in mortality group were significantly higher than those in surviving group. In addition, previous autopsy results have shown that pertussis deaths often involve multiple organ damage and dysfunction [31]. In this study, biochemical indicators related to organ function, such as NT-BNP, Cr, LDH, CK, and myoglobin, were higher in mortality group before ET than in surviving group. Before ET, blood gas analysis showed that PH and PO_2_ were lower in mortality group than in surviving group, while PCO_2_ and Lac were higher in mortality group than in surviving group. The incidences of heart failure, cardiogenic shock, and acute kidney injury in mortality group were higher than those in surviving group, suggesting that severe pertussis patients with pulmonary hypertension, pertussis encephalopathy, and organ dysfunction such as heart and kidney have a poor prognosis.

In addition to ET, all severe pertussis patients received comprehensive treatment, such as antibiotic therapy, mechanical ventilation, treatment for pulmonary hypertension, ECMO, immunoglobulin and hormone therapy, etc. Severe pertussis often leads to severe pneumonia and respiratory failure, which requires mechanical ventilation intervention [32]. Currently, there is no unified indication for mechanical ventilation. The treatment of pulmonary hypertension mainly uses inhaled NO, prostacyclin pathway agonists, etc. The overall survival rate of ECMO treatment is still low, and there is no consensus on the best time to start ECMO treatment for severe pertussis [33]. Glucocorticoid can alleviate symptoms to a certain extent, but their survival benefits for severe pertussis are still controversial [34]. A study by Hon et al. found that the proportion of hormone use in the mortality group was significantly higher than that in the surviving group [35]. In this study, the use rate of glucocorticoid, treatment for pulmonary hypertension and vasoactive drugs in the mortality group was higher than that in the surviving group, while there was no difference in other treatments such as mechanical ventilation, immunoglobulin and ECMO. This suggested that when general treatment for severe pertussis is ineffective and glucocorticoid, pulmonary hypertension drugs and vasoactive drugs are needed, the prognosis of the child may be poor.

Our study has some limitations based on survey data. This study is a single-center, retrospective study with a relatively small number of cases in the mortality group, making it impossible to conduct a Logistic regression analysis of risk factors. In the future, we will strive to conduct multi-center, large-sample, prospective studies, which will have more clinical significance.

## Conclusion

The mortality of severe pertussis is high and should not be ignored in clinical practice. ET is an effective treatment method to improve the prognosis of severe pertussis patients. The higher the peak of WBC, the more severe the pulmonary hypertension, and the more organs such as the brain, heart and kidneys are involved, the worse the prognosis. Early intervention with other treatments in addition to ET may be beneficial for improving the prognosis.

## Data Availability

The datasets used and/or analysed during the current study are available from the corresponding author on reasonable request.
